# A Comprehensive Literature Review on Managing Systemic Lupus Erythematosus: Addressing Cardiovascular Disease Risk in Females and Its Autoimmune Disease Associations

**DOI:** 10.7759/cureus.43725

**Published:** 2023-08-18

**Authors:** Saleha Dar, Sabina Koirala, Arooba Khan, Mounika Deepthi Bellary, Arya V Patel, Bejoi Mathew, Rahul Singh, Nahida Baigam, Waleed Razzaq, Zain U Abdin, Uzzam Ahmed Khawaja

**Affiliations:** 1 Department of Adult Medicine, Louisiana State University Health Sciences Center, Shreveport, USA; 2 Department of Medicine, Gandaki Medical College, Pokhara, NPL; 3 Department of Internal Medicine, Khyber Medical College, Peshawar, PAK; 4 Department of Medicine, Dr. YSR University of Health Sciences, Andhra Pradesh, IND; 5 Department of Internal Medicine, Smt. Nathiba Hargovandas Lakhmichand (NHL) Municipal Medical College, Ahmedabad, IND; 6 Department of Internal Medicine, Sri Devaraj Urs Medical College, Kolar, IND; 7 Department of Medicine, Armed Forces Medical College, Pune, IND; 8 Department of Medicine, Association of Physicians of Pakistani Descent of North America (APPNA), Westmont, USA; 9 Department of Internal Medicine, Services Hospital Lahore, Lahore, PAK; 10 Department of Medicine, District Head Quarter Hospital, Faisalabad, PAK; 11 Department of Pulmonary and Critical Care Medicine, Jinnah Medical and Dental College, Karachi, PAK; 12 Department of Clinical and Translational Research, Dr. Ferrer BioPharma, South Miami, USA

**Keywords:** antiphospholipid antibody syndrome, myasthenia gravis, therapeutic interventions, cardiac manifestations, systemic lupus erythematosus, adverse pregnancy outcomes, serum biomarkers

## Abstract

This review aimed to evaluate the mechanism of premature cardiovascular disease (CVD) in systemic lupus erythematosus (SLE) patients, particularly in the female population, and emphasize the need for early management interventions; explore the association between SLE and two autoimmune diseases, myasthenia gravis (MG) and antiphospholipid antibody syndrome (APS), and their management strategies; and evaluate the effectiveness of pharmacological and non-pharmacological interventions in managing SLE, focusing on premenopausal females, females of childbearing age, and pregnant patients. We conducted a comprehensive literature review to achieve these objectives using various databases, including PubMed, Google Scholar, and Cochrane. The collected data were analyzed and synthesized to provide an evidence-based overview of SLE, its management strategies as an independent disease, and some disease associations. The treatment should be focused on remission, preventing organ damage, and improving the overall quality of life (QOL). Extensive emphasis should also be focused on diagnosing SLE and concurrent underlying secondary diseases timely and managing them appropriately.

## Introduction and background

Systemic lupus erythematosus (SLE) can be defined as a chronic multisystem autoimmune disease with a wide range of systemic manifestations involving almost all organs and tissues with its effects varying from minor cutaneous involvement to major organ damage. The burden of SLE and its survival rates vary across Asia-Pacific countries, with crude incidence rates (100,000 per year) ranging from 0.9 to 3.1, while prevalence rates range from 4.3 to 45.3 (per 100,000). A higher rate of renal involvement, which is one of the main systems involved in death, was reported for Asians as compared to whites [1]. Most of the pathology in SLE is due to immune complex (IC) deposition in various organs, which leads to the activation of complement and other mediators of inflammation. The symptoms of SLE vary widely. Fatigue in SLE is multifactorial [2]. SLE attacks the immune system, which, in addition to the immunosuppressive drugs used to treat the disease, reduces the body’s ability to fight infections [3]. General symptoms include weight loss, appetite loss, headache, myalgias, arthralgias, fever, and malaise. Weight gain may also happen due to corticosteroid treatment or nephrotic syndrome anasarca [4]. Involvement of the musculoskeletal system is common in patients with SLE [5]. Arthritis, arthralgia, osteonecrosis, and myopathy are the main manifestations [6]. Dermatological manifestations include a malar rash, which comprises an erythematous rash over the cheeks and nasal bridge.

Other features include photosensitivity, discoid rash, and alopecia. Telangiectasias, urticaria, vasculitic purpura, bullous lesions, panniculitis, livedo reticularis (LR), and Raynaud’s phenomenon are features that are related to but not specific to SLE. Deposits of immunoglobulins are found in all cases of SLE, but only 50% lead to nephritis [2]. Sepsis and renal failure are two main causes of death in patients with SLE, and the kidney is the most common organ involved. Glomerular disease usually manifests in the first few years and is usually asymptomatic. Symptoms of uremia and fluid overload may be caused by acute or chronic renal failure. Nephritic syndrome may present as hematuria and hypertension, while nephrotic syndrome may present as edema, weight gain, or hyperlipidemia. Lupus nephritis is one of the most common manifestations of SLE and one of the most damaging too. It is caused by the deposition of immune complexes and occurs in more than 50% of SLE patients [7]. Of patients with SLE, 25%-75% report neurological involvement [8]. SLE may cause status epilepticus, aseptic meningitis, optic neuropathy, myelopathy, and transverse myelitis with spastic paraparesis [7]. Stroke and transient ischemic attack may be related to antiphospholipid antibody syndrome (APS) or vasculitis. Migraines may be related to APS [9]. Headache and mood disorders might be the most common neurological manifestations of SLE [10,11]. Serositis can affect both the cardiac and pulmonary systems, and pleurisy with pleuritic chest pain with or without pleural effusion is the most common pulmonary manifestation of SLE [2]. Alveolar hemorrhage, pulmonary hypertension (PH), and thromboembolic disease are some of the other effects of SLE on the pulmonary system [2]. Oral ulcers are common in SLE, and abdominal pain may signify peritonitis, pancreatitis, mesenteric ischemia, and vasculitis. Autoimmune hepatitis leading to jaundice can also occur [7].

The most common cardiac presentation of SLE is pericarditis. Myocarditis leading to heart failure can also occur. Libman-Sacks endocarditis is also a manifestation of SLE, while coronary vasculitis presenting as angina can occur rarely. Accelerated ischemic heart disease is also commonly associated with SLE. Vasculitis, peripheral vascular disease, digital ulcers, livedo reticularis, and Raynaud’s phenomenon are common vascular manifestations of SLE [3]. Conjunctivitis, interstitial keratitis, episcleritis, and diffuse or nodular scleritis can occur with SLE, with keratoconjunctivitis sicca being the most common ocular manifestation [12,13]. SLE is associated with worse pregnancy outcomes with an increased risk of fetal death in utero, spontaneous abortions, and fetal retardations [8]. Neonatal lupus affects 3% of babies born to mothers with SLE [4]. Patients with SLE suffer from thyroid dysfunction more commonly than the general population [14]. The rate of fractures in lupus is five times higher than in the general population [15]. Glucocorticoid use can suppress pituitary function, while vitamin D deficiency is common due to avoidance of sun exposure [16]. Cytopenias such as leukopenia, lymphopenia, anemia, or thrombocytopenia can be seen in SLE, while leukopenia and lymphopenia are more common. ESR is frequently elevated in active disease, and plasma homocysteine levels are considered a risk factor for stroke in SLE [2].

Diagnosis and classification criteria

SLE diagnosis can be challenging given a wide variety of clinical features with the potential for multiple organ system involvement, as well as the overlap of clinical features with other autoimmune conditions. Initial evaluation requires a comprehensive history and physical examination along with a careful interpretation of relevant laboratory testing. It is also important to know that there are no diagnostic criteria established for the diagnosis of SLE so far. Current practices, therefore, rely on classification criteria to identify SLE cases. The 2019 European League Against Rheumatism/American College of Rheumatology (EULAR/ACR) classification criteria [17,18] for systemic lupus erythematosus has improved sensitivity (96.1%) and specificity (93.4%) compared to previous American College of Rheumatology (ACR) 1997 [19] and Systemic Lupus International Collaborating Clinics (SLICC) 2012 criteria [20]. Table 1 demonstrates the improvement in specificity from the previous ACR 1997 while maintaining the sensitivity of SLICC 2012 criteria.

**Table 1:** Operating characteristics of new EULAR/ACR 2019 criteria compared to ACR 1997 and SLICC 2012 classification criteria. ACR, American College of Rheumatology; SLICC, Systemic Lupus International Collaborating Clinics; CI, confidence intervals; EULAR, European League Against Rheumatism Reproduced under the terms of the Creative Commons attribution license: Aringer M, Costenbader K, Daikh D, et al.: 2019 European League Against Rheumatism/American College of Rheumatology classification criteria for systemic lupus erythematosus. Ann Rheum Dis. 2019, 78:1151-9. 10.1136/annrheumdis-2018-214819 [17] and Aringer M, Costenbader K, Daikh D, et al.: 2019 European League Against Rheumatism/American College of Rheumatology classification criteria for systemic lupus erythematosus. Arthritis Rheumatol. 2019, 71:1400-12. 10.1002/art.40930 [18]

	ACR 1997 criteria	SLICC 2012 criteria	EULAR/ACR 2019 criteria
Derivation			
Sensitivity (95% CI)	0.85 (0.81-0.88)	0.97 (0.95-0.98)	0.98 (0.97-0.99)
Specificity (95% CI)	0.95 (0.93-0.97)	0.90 (0.87-0.92)	0.96 (0.95-0.98)
Combined (95% CI)	1.80 (1.76-1.83)	1.87 (1.84-1.90)	1.94 (1.92-1.96)
Validation			
Sensitivity (95% CI)	0.83 (0.80-0.85)	0.97 (0.95-0.98)	0.96 (0.95-0.98)
Specificity (95% CI)	0.93 (0.91-0.95)	0.84 (0.80-0.87)	0.93 (0.91-0.95)
Combined (95% CI)	1.76 (1.73-1.80)	1.80 (1.77-1.84)	1.90 (1.87-1.92)

**Figure 1:**
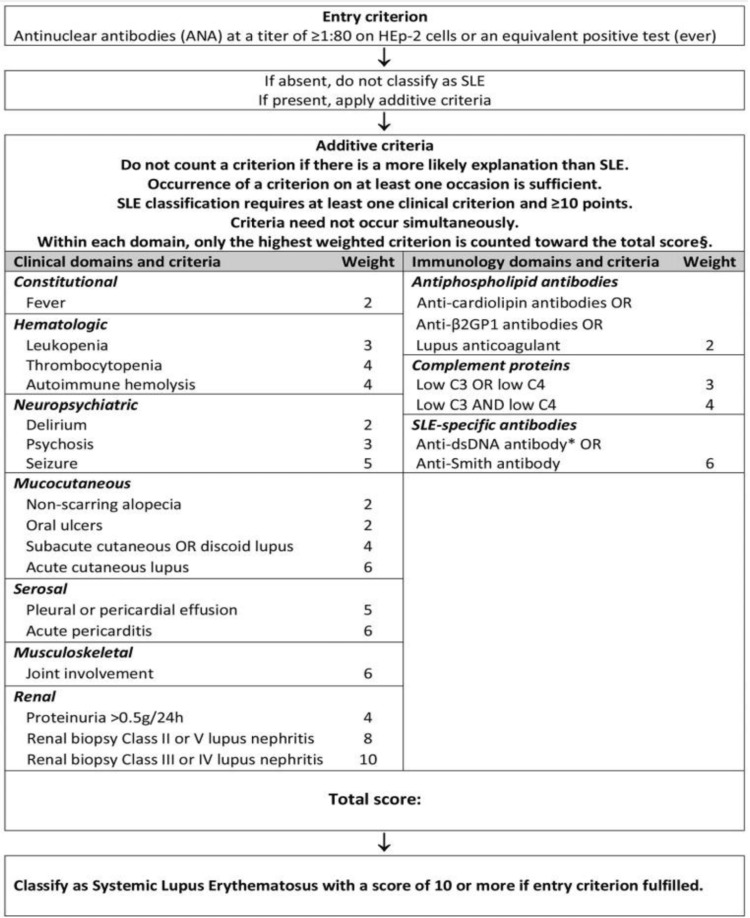
Classification criteria for systemic lupus erythematosus. ANA, antinuclear antibody; SLE, systemic lupus erythematosus; anti-β2GP1, anti-β-2-glycoprotein 1; anti-dsDNA, anti-double-stranded DNA Reproduced under the terms of the Creative Commons attribution license: Aringer M, Costenbader K, Daikh D, et al.: 2019 European League Against Rheumatism/American College of Rheumatology classification criteria for systemic lupus erythematosus. Ann Rheum Dis. 2019, 78:1151-9. 10.1136/annrheumdis-2018-214819 [17] and Aringer M, Costenbader K, Daikh D, et al.: 2019 European League Against Rheumatism/American College of Rheumatology classification criteria for systemic lupus erythematosus. Arthritis Rheumatol. 2019, 71:1400-12. 10.1002/art.40930 [18]

The recent classification designates antinuclear antibody (ANA) positivity as the qualifying factor to enter the SLE criteria given its high sensitivity. It also emphasizes the concept that all criteria must only be taken into account if there is no alternative cause suspected and the manifestation is most likely explained by SLE. Moreover, the presence of at least one clinical criterion is a must along with 10 or more points to classify as having SLE. Figure 1 discusses these points further along with a mention of clinical and immunologic criteria that are central to this classification.

While the sensitivity of the most recent EULAR/ACR classification criteria is 96%-99%, it should be kept in mind that about 1%-4% of cases of SLE can be missed if the classification is strictly followed for the purpose of diagnosis. We must also keep in mind that while the classification encompasses several clinical and immunologic manifestations of the disease, it does not incorporate various other and less common features [21]. This is particularly pertinent to diagnosing patients with ANA-negative disease [22,23]. Table 2 summarizes other features relevant to the diagnosis of a disease that is not included in the EULAR/ACR 2019 criteria.

**Table 2:** Organ domains and relevant features included/not included in the EULAR/ACR 2019 criteria. ISN, International Society of Nephrology; RPS, Renal Pathology Society; ACR, American College of Rheumatology; EULAR, European League Against Rheumatism; ACLE, acute cutaneous lupus erythematosus; SCLE, subacute cutaneous lupus erythematosus; DLE, discoid lupus erythematosus; ANA, antinuclear antibody; APS, antiphospholipid syndrome Reproduced under the terms of the Creative Commons attribution license: Aringer M, Johnson SR: Classifying and diagnosing systemic lupus erythematosus in the 21st century. Rheumatology (Oxford). 2020, 59:v4-v11. 10.1093/rheumatology/keaa379 [21]

Domain	EULAR/ACR 2019 classification criteria	Other features relevant for SLE diagnosis
Autoantibodies		
1	ANA (obligatory entry criterion) Anti-Sm: 6, anti-dsDNA (highly specific test): 6	Anti-Ro/anti-La, anti-U1RNP, anti-dsDNA (tests of lesser specificity), anti-nucleosome/anti-chromatin, anti-histone, anti-C1q, anti-ribosomal P, positive Coombs test without hemolysis, false-positive serology for syphilis
2	Anti-cardiolipin (medium to high titer): 2, anti-β-2-glycoprotein 1: 2, lupus anticoagulant: 2	
Complement		
3	C3 and C4 low: 4, C3 or C4 low: 3	CH50 low, complement split products on erythrocytes
Mucocutaneous manifestations		
4	ACLE: 6, SCLE: 4, DLE: 4, oral ulcers: 2, non-scarring alopecia: 2	Lupus tumidus, lupus panniculitis/lupus profundus, chilblains lupus, leukocytoclastic vasculitis, urticarial vasculitis, nasal ulcers
Lupus nephritis		
5	ISN/RPS class III or IV nephritis: 10, ISN/RPS class II or V nephritis: 8, proteinuria >0.5 g/day: 4	IgA nephritis, cellular casts
Musculoskeletal manifestations		
6	Joint involvement: 6	Myositis
Serositis		
7	Acute pericarditis: 6, pleural or pericardial effusion: 5	Sterile peritonitis
Neuropsychiatric manifestations		
8	Seizure: 5, psychosis: 3, delirium: 2	(Transverse) myelitis (often APS-related), chorea, mononeuritis multiplex, cranial neuropathy, peripheral neuropathy, lupus headache
Hematological manifestations		
9	Thrombocytopenia: 4, autoimmune hemolytic anemia: 4, leukopenia: 3	Thrombotic thrombocytopenic purpura, other forms of hemolytic anemia, anemia of chronic disease, lymphopenia
Constitutional symptoms		
10	Fever:2	Arthralgias, myalgias, fatigue, lymphadenopathy
Other uncommon SLE organ manifestations		
		Pneumonitis, interstitial lung disease, pulmonary artery hypertension, Libman-Sacks endocarditis (APS-related), myocarditis, hepatitis, pancreatitis. gastrointestinal vasculitis, interstitial cystitis

Hence, making the diagnosis through an individualized approach is of utmost importance to avoid excluding potential SLE patients from receiving appropriate therapies [24].

Treatment of SLE in general

Immunomodulators

Hydroxychloroquine (HCQ): Hydroxychloroquine (HCQ) modulates the immune response pleiotropically by inhibiting B-cell receptor and Toll-like receptor (TLR) signaling, as well as intracellular TLR-3 and TLR-7 activation, which is important in nucleic acid sensing [25,26]. It raises lysosomal pH, interfering with major histocompatibility complex (MHC) antigen binding and thus processing autoantigens and cytokine secretions [27]. By interfering with the stimulator of interferon gene (STING) pathway, HCQ has an anti-type 1 interferon effect [28]. Unless there is a clear contraindication, HCQ is recommended for all patients with SLE. It is the only medication that has been shown to increase survival in lupus patients [29,30]. It has been shown to reduce lupus flares [31], prevent organ damage [32] including cardiovascular events [33] and triple mycophenolate response in lupus nephritis [34], prevent seizures [35,36], and lower the risk of developing neuropsychiatric lupus [37]. HCQ alleviates skin manifestations [38,39] and arthritis [40]. HCQ improves lipids [27], lowers insulin resistance [41] and the risk of thrombosis [42,43], and improves bone density [44].

**Figure 2:**
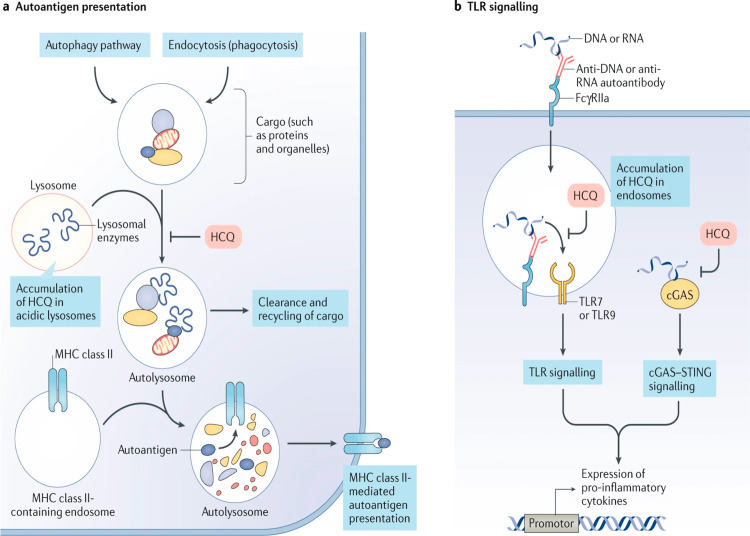
Mechanism of action of hydroxychloroquine. (a) HCQ enters and accumulates in lysosomes along a pH gradient. In lysosomes, hydroxychloroquine inhibits the degradation of cargo derived externally (via endocytosis or phagocytosis) or internally (via the autophagy pathway) in autolysosomes by increasing the pH to prevent the activity of lysosomal enzymes. Inhibition of lysosomal activity can prevent MHC class II-mediated autoantigen presentation. (b) Hydroxychloroquine can also accumulate in endosomes and bind to the minor groove of double-stranded DNA. This drug can inhibit TLR signaling by altering the pH of endosomes (involved in TLR processing) and/or preventing TLR-7 and TLR-9 from binding their ligands (RNA and DNA, respectively). Hydroxychloroquine can also inhibit the activity of the nucleic acid sensor cGAS by interfering with its binding to cytosolic DNA. By preventing TLR signaling and cGAS-STING signaling, hydroxychloroquine can reduce the production of pro-inflammatory cytokines, including type I interferons. HCQ, hydroxychloroquine; MHC, major histocompatibility complex; DNA, deoxyribonucleic acid; TLR, Toll-like receptor; RNA, ribonucleic acid; cGAMP, cyclic GMP-AMP; cGAS, cGAMP synthase; STING, stimulator of interferon genes Reproduced under the terms of the Creative Commons attribution license: Schrezenmeier E, Dörner T: Mechanisms of action of hydroxychloroquine and chloroquine: implications for rheumatology. Nat Rev Rheumatol. 2020, 16:155-66. 10.1038/s41584-020-0372-x [51]

HCQ has no immunosuppressive properties and does not increase the risk of infection or cancer [45,46]. Retinal toxicity is a rare complication that becomes more common after 20 years of treatment [47]. Retinal screening is done at the beginning, at five years, and then every year [48]. The screening test of choice is optical coherence tomography [49]. Hyperpigmentation is possible, while cardiomegaly and myopathy are two extremely rare complications. The daily dose should not exceed the threshold of 5 mg/kg real body weight based on existing evidence, which suggests that the risk of toxicity is very low for doses less than 5 mg/kg real body weight. It is worth noting that the efficacy of HCQ in lupus has been established in studies with a prescribed dose of 6.5 mg/kg/day, so it remains to be seen whether a lower dose will have comparable clinical effects. Patients in long-term remission may have their dose reduced, although no formal studies have addressed this strategy. In patients with cutaneous manifestations and HCQ-induced retinal toxicity, quinacrine, an alternative antimalarial, may be considered [50]. Figure 2 illustrates the mechanism of action of HCQ.

**Figure 3:**
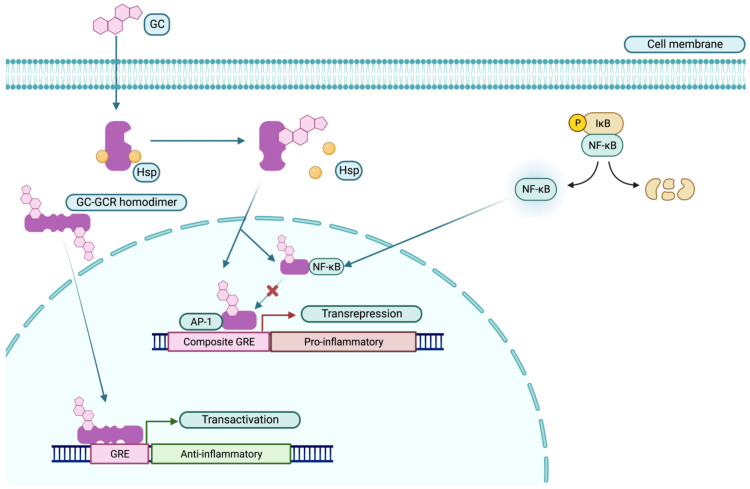
Genomic mechanisms of glucocorticoid-induced anti-inflammation. GCs bind to their cytosolic GCR, which subsequently loses its chaperoning proteins, such as Hsp. Homodimers are formed, travel to the nucleus, bind to the GRE, and upregulate the expression of certain genes (e.g., lipocortin-1 and genes involved in metabolism), a mechanism called transactivation. mGC-GCR can bind to transcription factors such as AP-1 and NF-kβ, inhibiting the transcription of their target genes (e.g., IL-2 and TNF-α) by a mechanism called transrepression. Further, direct binding of mGC-GCR alongside AP-1 on composite GREs leads to transrepression. GC, glucocorticoid; GCR, glucocorticoid receptor; Hsp, heat shock proteins; GRE, glucocorticoid response element; mGC-GCR, monomeric GC-GCR complex; AP-1, activator protein 1; NF-kβ, nuclear factor kappa β; IL-2, interleukin-2; TNF-α, tumor necrosis factor-α Reproduced under the terms of the Creative Commons attribution license: Téllez Arévalo AM, Quaye A, Rojas-Rodríguez LC, Poole BD, Baracaldo-Santamaría D, Tellez Freitas CM: Synthetic pharmacotherapy for systemic lupus erythematosus: potential mechanisms of action, efficacy, and safety. Medicina (Kaunas). 2022, 59:10.3390/medicina59010056 [62]

Corticosteroids: By non-selectively decreasing the expression of adhesion molecules and cytokines (such as interleukin (IL)-2, IL-6, tumor necrosis factor-α (TNF-α), and prostaglandins), corticosteroids have strong anti-inflammatory and immunosuppressive effects. The initial to long-term goal should be to reduce the daily dose to less than 7.5 mg/day of prednisone equivalent or to stop them because long-term glucocorticoid therapy can have several adverse effects, including irreversible organ damage [52-55]. Continuous glucocorticoid doses > 7.5 mg/day carry a markedly increased risk, and some studies even indicated a lower dose as potentially harmful [56-59]. As a result, the management strategy is to use corticosteroids as bridging therapy (oral or intramuscular (IM)) as part of an induction regimen or to treat an acute flare rather than as a maintenance treatment [60]. After ruling out infections, high-dose intravenous (IV) methylprednisolone (typically 250-1,000 mg/day for three days) is frequently used in acute, organ-threatening diseases (such as renal and neuropsychiatric) [61]. Figure 3 illustrates the genomic mechanisms of glucocorticoid-induced anti-inflammation.

Immunosuppressants

Methotrexate: Methotrexate is an antimetabolite that prevents the synthesis of purines by irreversibly binding to the enzyme dihydrofolate reductase and thus inhibits DNA synthesis, repair, and replication. Although the mechanism of its anti-inflammatory effects is not fully understood, it goes beyond arresting the cell cycle through folate depletion. For instance, co-administration of folate reduces side effects while not impairing its efficacy. Reduced circulating pro-inflammatory T cells, increased anti-inflammatory adenosine signaling, apoptosis of activated lymphocytes, reduced reactive oxygen species, and decreased adhesion molecules on endothelial and synovial cells are some pleiotropic effects of low-dose methotrexate [63]. Methotrexate (15-20 mg per week) was effective in treating cutaneous and articular disease, according to Carneiro and Sato [64], and it made it easier to lower the dosage of prednisone. A steroid-sparing effect was demonstrated by Fortin et al. [65], along with a decline in disease activity. A 60% improvement in the joint count was discovered by Rahman et al. [66]. Methotrexate is an effective and widely used treatment for cutaneous disease resistant to antimalarial and topical medications [67]. However, because it is a teratogen, methotrexate cannot be used during pregnancy.

Cyclophosphamide (CYC): A highly toxic alkylating agent, cyclophosphamide suppresses the production of antibodies by depleting T and B cells [68]. Cyclophosphamide (CYC) should only be used as a last resort in non-major organ manifestations that are refractory to other treatments and should only be considered in organ-threatening diseases (renal, cardiopulmonary, or neuropsychiatric). Due to its gonadotoxic effects, it should be used with caution in females and males of fertile age [69-71]. In premenopausal patients with SLE, concurrent use of gonadotropin hormone-releasing hormone (GnRH) analogs reduces the ovarian reserve reduction brought on by CYC therapy [72-74]. Prior to starting treatment, it is important to provide information about the potential for ovarian cryopreservation. Infections and other risks associated with CYC therapy, such as cancer, should also be taken into account [75,76].

**Figure 4:**
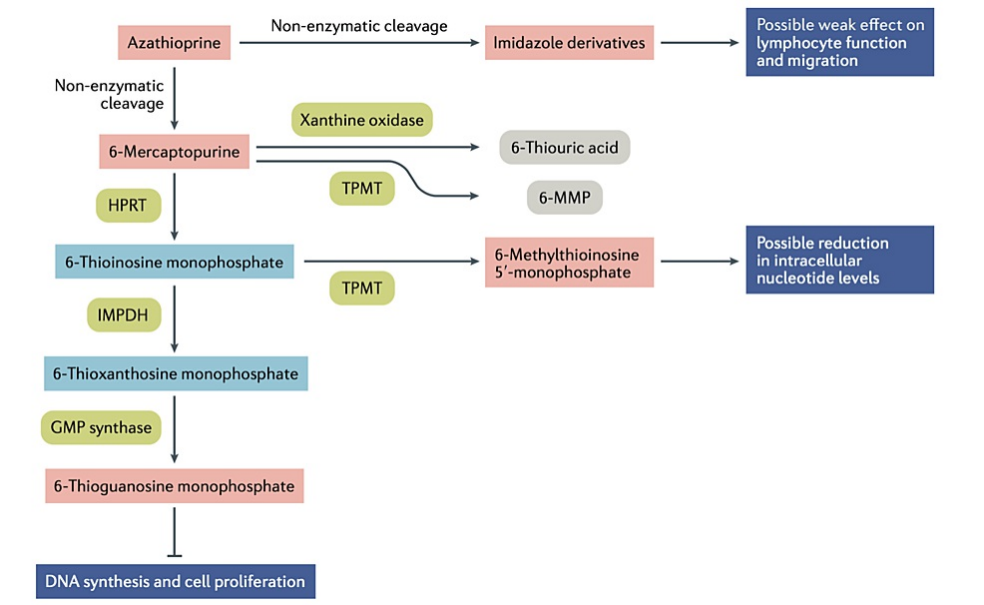
Immunomodulatory effects of azathioprine. Azathioprine is cleaved non-enzymatically into either 6-mercaptopurine or imidazole derivatives (such as mercapto-imidazole) by sulfhydryl-containing compounds; these imidazole derivatives are thought to have weak immunomodulatory effects. 6-Mercaptopurine is further metabolized by xanthine oxidase and TPMT into 6-thiouric acid and 6-MMP, respectively, which are both non-toxic. In a stepwise manner, 6-mercaptopurine can also be metabolized to toxic 6-thioguanosine nucleotides, which inhibit effective DNA synthesis and cell proliferation. Firstly, 6-mercaptopurine is converted to 6-thioinosine monophosphate by HPRT; 6-thioinosine monophosphate is metabolized to 6-thioxanthosine monophosphate by IMPDH, which is subsequently converted to 6-thioguanosine monophosphate by GMP synthase. Inhibition of cell proliferation is the main immunosuppressive effect of azathioprine on lymphocytes. 6-Thioinosine monophosphate can also be metabolized by TPMT to 6-methylthioinosine 5′-monophosphate, which is thought to reduce the availability of nucleotides in cells. TPMT, thiopurine S-methyltransferase; 6-MMP, 6-methyl mercaptopurine; DNA, deoxyribonucleic acid; HPRT, hypoxanthine-guanine phosphoribosyltransferase; IMPDH, inosine-5′monophosphate-dehydrogenase; GMP, guanosine monophosphate Reproduced under the terms of the Creative Commons attribution license: Broen JC, van Laar JM: Mycophenolate mofetil, azathioprine and tacrolimus: mechanisms in rheumatology. Nat Rev Rheumatol. 2020, 16:167-78. 10.1038/s41584-020-0374-8 [82]

Azathioprine: Azathioprine, a purine analog, is inactive until it is metabolized by the liver and erythrocytes into mercaptopurine, at which point it inhibits DNA synthesis and prevents immune system cell proliferation. There is a high prevalence of gastrointestinal tract toxicity, oral ulcers, nausea, vomiting, diarrhea, and epigastric pain. Leukopenia and, less frequently, thrombocytopenia and anemia are the results of dose-related toxicity to the bone marrow [77]. Compared to corticosteroids alone, azathioprine has been shown in two small randomized studies to decrease mortality, the frequency of flare-ups, and corticosteroid use, including in patients with severe renal or central nervous system (CNS) disease [78,79]. Azathioprine is frequently used as a corticosteroid-sparing agent in extra-renal lupus [80]. As the metabolite 6-methyl mercaptopurine (6-MMP) is not produced in the developing fetus, azathioprine is still a great option for controlling renal and extra-renal disease during pregnancy [81]. Figure 4 illustrates the immunomodulatory effects of azathioprine.

**Figure 5:**
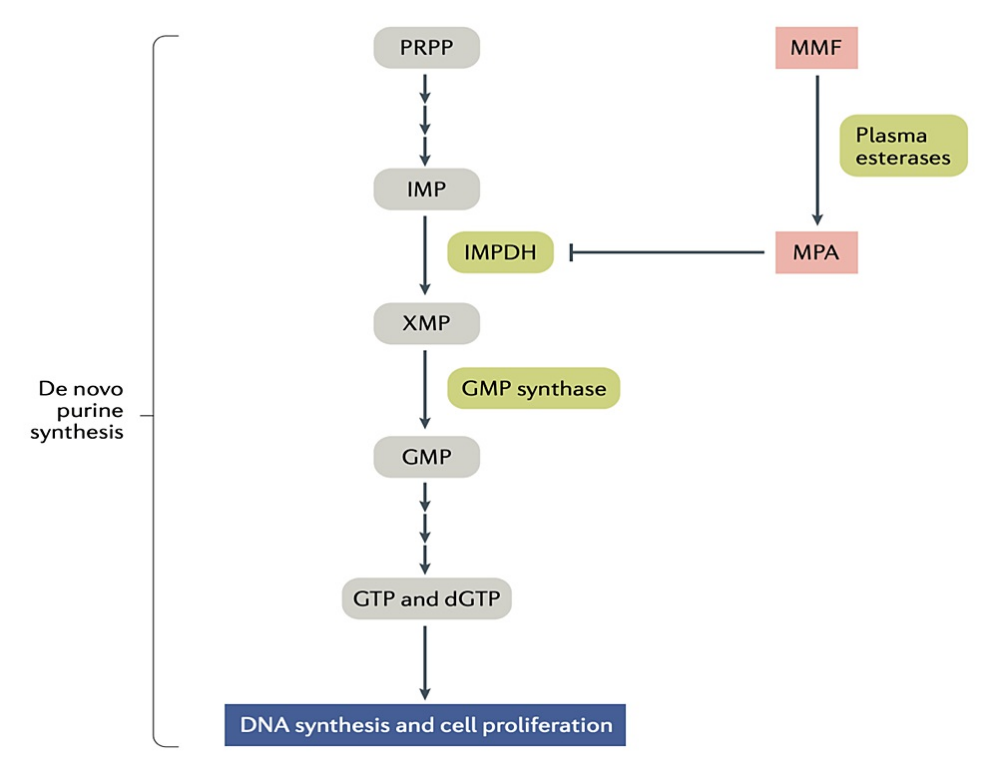
Inhibition of nucleotide synthesis by mycophenolate mofetil. MMF is metabolized into MPA by carboxylesterases; the latter inhibits the synthesis of guanine nucleotides through the de novo purine synthesis pathway. This pathway begins with the conversion of 5-ribose phosphate to PRPP. PRPP is subsequently converted to IMP, which is dehydrogenated to XMP by IMPDH and subsequently dehydrogenated to GMP by GMP synthase. GMP is converted to GTP and dGTP, which are needed for DNA synthesis. MPA is a strong inhibitor of IMPDH, and the inhibition of IMPDH leads to low availability of nucleotides (GMP, GTP, and dGTP) and hence prevents DNA replication and subsequently cell proliferation. MMF, mycophenolate mofetil; MPA, mycophenolic acid; PRPP, 5-phosphoribosyl-1-pyrophosphate; IMP, inosine monophosphate; XMP, xanthine monophosphate; IMPDH, inosine monophosphate dehydrogenase; GMP, guanosine monophosphate; GTP, guanosine triphosphate; dGTP, deoxyguanosine triphosphate; DNA, deoxyribonucleic acid Reproduced under the terms of the Creative Commons attribution license: Broen JC, van Laar JM: Mycophenolate mofetil, azathioprine and tacrolimus: mechanisms in rheumatology. Nat Rev Rheumatol. 2020, 16:167-78. 10.1038/s41584-020-0374-8 [82]

Mycophenolate mofetil (MMF): The rate-limiting enzyme in the synthesis of guanosine nucleotides, inosine monophosphate dehydrogenase, is inhibited by mycophenolate mofetil, a prodrug of mycophenolic acid, an effective immunosuppressant for both renal and non-renal lupus (although not in neuropsychiatric disease) [83-85]. Enteric-coated mycophenolate sodium (EC-MPS) outperformed azathioprine in a recent randomized, open-label trial for extra-renal SLE in terms of achieving remission and lowering flares [86]. However, because of its teratogenic potential, it needs to be discontinued at least six weeks prior to conception, and a higher price compared to azathioprine or methotrexate limits its ability to be recommended universally in females of reproductive age with non-renal manifestations. Figure 5 illustrates the inhibition of nucleotide synthesis by mycophenolate mofetil.

**Figure 6:**
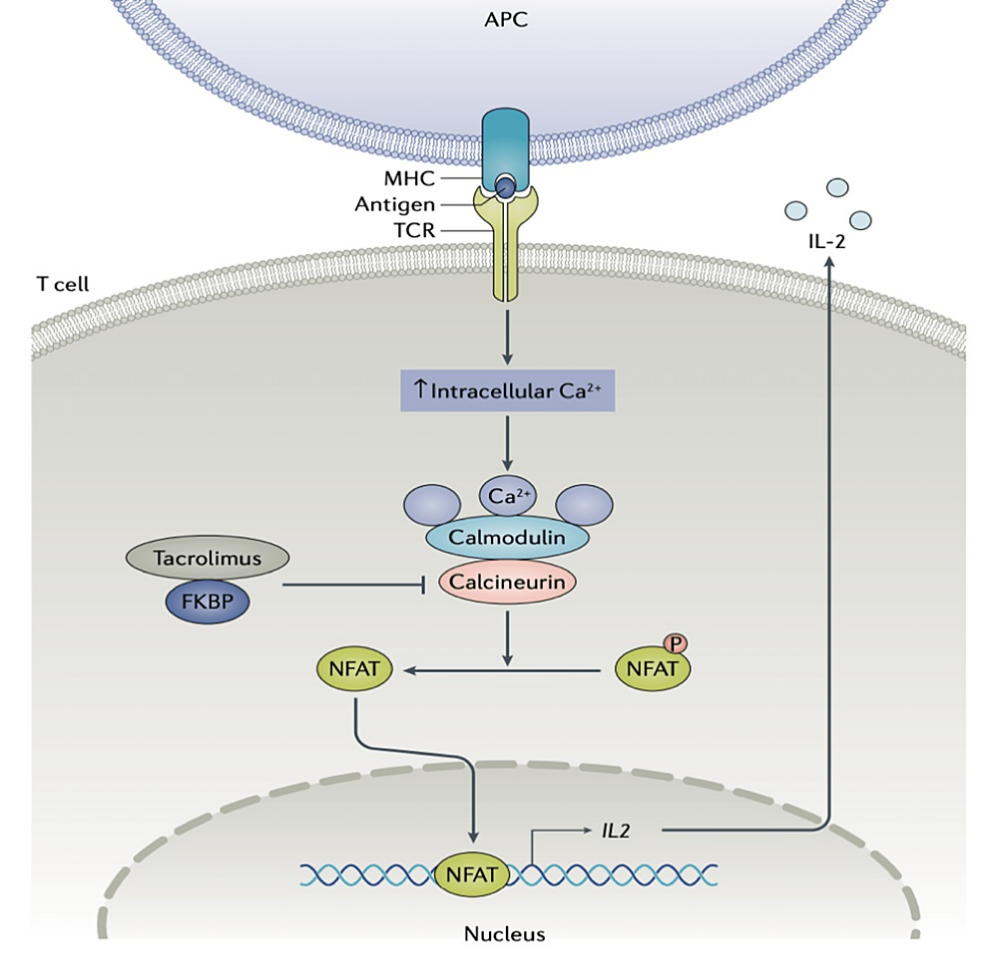
Inhibition of T cells by tacrolimus. In T cells, TCR signaling increases levels of calcium in the cytoplasm, which leads to the activation of the calcineurin-NFAT pathway. In this pathway, dephosphorylation of NFAT leads to the activation and nuclear translocation of NFAT and the transcription of IL-2. Tacrolimus binds to FKBP, and this tacrolimus-FKBP complex suppresses the activation of the calcineurin-NFAT pathway, leading to a reduction in IL-2 production and inhibiting early activation of T cells. TCR, T-cell receptor; NFAT, nuclear factor of activated T cell; IL-2, interleukin-2; FKBP, FK506-binding protein; APC, antigen-presenting cell; MHC, major histocompatibility complex Reproduced under the terms of the Creative Commons attribution license: Broen JC, van Laar JM: Mycophenolate mofetil, azathioprine and tacrolimus: mechanisms in rheumatology. Nat Rev Rheumatol. 2020, 16:167-78. 10.1038/s41584-020-0374-8 [82]

Calcineurin inhibitors: Calcineurin inhibitors block T cells through the inhibition of calcineurin [76]. This inhibits T cells and lowers levels of IL-1b, interferon-gamma (IFN-γ), IL-6, and IL-10 by preventing transcription factors such as nuclear factor of activated T cells (NFAT) from translocating. B-cell activation is also compromised, in addition to class switching and immunoglobulin production. Furthermore, calcineurin inhibitors stabilize podocytes and decrease mesangial proliferation in the kidneys, improving proteinuria [87-89]. Tacrolimus is preferred for SLE. Tacrolimus has been shown to be additive to mycophenolate mofetil for the treatment of lupus nephritis in particular [90] and has been studied as a monotherapy and as a component of a multi-targeted approach [90-92]. Other studies have shown that tacrolimus is a suitable substitute for mycophenolate mofetil [93]. A meta-analysis found that tacrolimus was superior to CYC for lupus nephritis induction therapy [94]. Tacrolimus is a treatment option for refractory cutaneous disease during pregnancy [95] and can be applied topically. Figure 6 illustrates the inhibition of T cells by tacrolimus.

Biological Agents

Belimumab: In SLE, irregular B-cell pathways are usually present. B cells play a role in autoantibody production, T-cell antigen presentation, and cytokine release (including interferon-α, IL-6, IL-10, B-cell activating factor (BAFF), TNF-α, and a proliferation-inducing ligand (APRIL)) [96]. Belimumab, a fully humanized monoclonal antibody, binds to soluble BAFF, resulting in a decrease in the number of peripheral naive and transitional activated B cells [97,98]. Belimumab should be considered in extra-renal disease with insufficient control (ongoing disease activity or frequent flares) to first-line treatments (typically a combination of HCQ and prednisone with or without immunosuppressive agents) and an inability to taper glucocorticoid daily dose to acceptable levels (i.e., maximum of 7.5 mg/day). Belimumab may benefit patients with persistent disease; patients with high disease activity (e.g., Systemic Lupus Erythematosus Disease Activity Index (SLEDAI) > 10), prednisone dose > 7.5 mg/day, and serological activity (low C3/C4 and high anti-dsDNA titers), with cutaneous, musculoskeletal, and serological manifestations, are more likely to respond [99-101].

**Figure 7:**
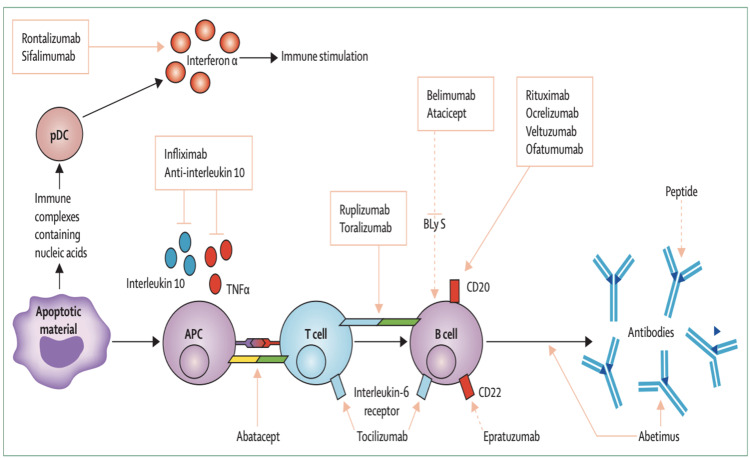
Targeted biological agents available and in present or previous clinical trials of systemic lupus erythematosus. pDC, plasmacytoid dendritic cell; BLyS, B lymphocyte stimulator; TNF-α, tumor necrosis factor-α; APC, antigen-presenting cell Reproduced under the terms of the Creative Commons attribution license: Murphy G, Lisnevskaia L, Isenberg D: Systemic lupus erythematosus and other autoimmune rheumatic diseases: challenges to treatment. Lancet. 2013, 382:809-18. 10.1016/S0140-6736(13)60889-2 [110]

Rituximab (RTX): Rituximab (RTX) is a chimeric monoclonal antibody that kills mature B cells and B-cell precursors by attacking CD20 on B cells. Due to the negative findings of randomized controlled trials (RCTs), RTX is currently only used off-label in patients with severe renal or extra-renal (primarily hematological and neuropsychiatric) disease that is resistant to other immunosuppressive agents and/or belimumab or in patients who have contraindications to these medications. More than one immunosuppressive drug must have failed before RTX is administered [102-105], with the possible exception of severe autoimmune thrombocytopenia (ITP) and hemolytic anemia, for which RTX has shown efficacy in both lupus patients and those with isolated immune thrombocytopenia (ITP) [106-108]. In lupus nephritis, RTX is typically considered when first-line therapies (CYC and MMF) have failed or when the condition is relapsing [102,109]. Other therapies such as anifrolumab, baricitinib, and atacicept are under study. Figure 7 illustrates targeted biological agents available in present or previous clinical trials of systemic lupus erythematosus.

**Figure 8:**
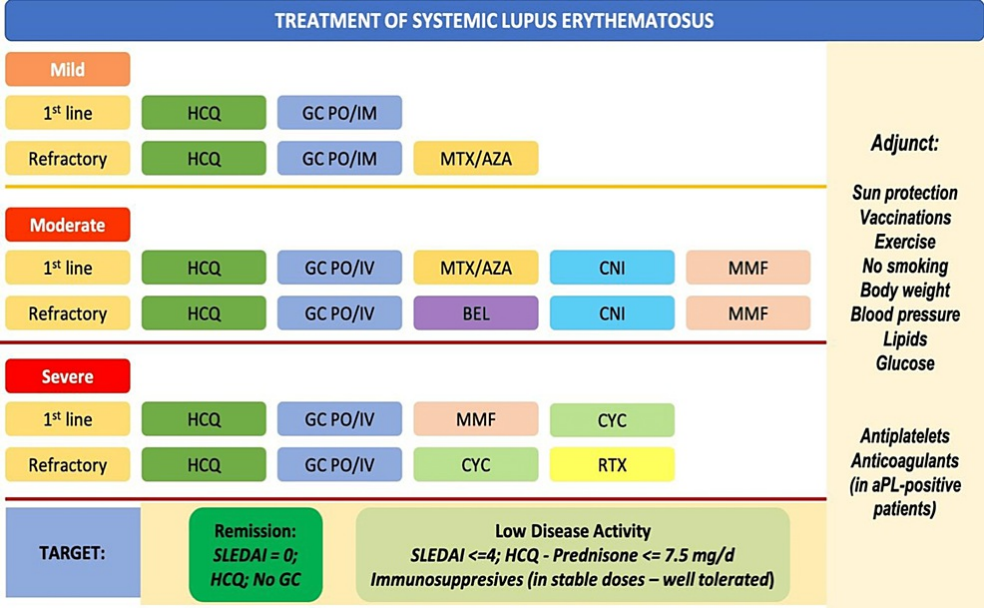
Management of SLΕ drugs, treatment strategy, targets of therapy, and adjunct therapy. Determination of severity in SLE is based on (a) the involvement of major organs or organ-threatening disease, (b) concomitant activity from multiple non-major organs, and (c) the need for the use of high doses of glucocorticoids and/or immunosuppressive therapy. SLE, systemic lupus erythematosus; aPL, antiphospholipid antibody; AZA, azathioprine; BEL, belimumab; CNI, calcineurin inhibitors; CYC, pulse cyclophosphamide; EULAR, European League Against Rheumatism; GC, glucocorticoids; PO, per oral; IM, intramuscular; IV, intravenous; MTX, methotrexate; HCQ, hydroxychloroquine; MMF, mycophenolate mofetil; RTX, rituximab; SLEDAI, SLE Disease Activity Index Reproduced under the terms of the Creative Commons attribution license: Fanouriakis A, Tziolos N, Bertsias G, Boumpas DT: Update οn the diagnosis and management of systemic lupus erythematosus. Ann Rheum Dis. 2021, 80:14-25. 10.1136/annrheumdis-2020-218272 [111]

Figure 8 illustrates the overview of the management of SLE based on the severity of the disease.

## Review

Managing SLE in the premenopausal period, females of childbearing age, and pregnancy

Recent SLE epidemiological studies report the global prevalence of SLE to be 15.87-108.92 per 100,000 persons, with 1.4-15.13 new cases per 100,000 persons per year [112]. Studies show that the disease prevalence and incidence are several folds in females compared to males [112,113] with a prevalence ratio ranging between 7:1 and 15:1 between females of childbearing age and males [114,115]. Given that SLE affects females of reproductive age, understanding issues regarding reproductive health including fertility, family planning, contraception, pregnancy implications, drug safety during pregnancy, and breastfeeding is of utmost importance.

Reproductive Ability 

Several risk factors play a role in decreasing fertility in females with SLE [116]. Chronic disease activity and recurrent flares diminish ovarian reserves by creating a state of inflammation within the ovaries and disturbing the hypothalamic pituitary ovarian axis [117-119]. A cross-sectional study also demonstrated an association between premature ovarian failure and specific SLE-related autoantibodies as well as different immunosuppressants used to treat the disease [120].

Management of SLE in Pregnant Mothers

For a long time, doctors advised females with SLE to put off having children out of fear of a disease flare or a bad outcome for the fetus. Having patients under good control on drugs suitable for pregnancy before conception is a crucial part of managing SLE during pregnancy. Patients should preferably be cared for by a multidisciplinary team with expertise in rheumatic illnesses and maternal-fetal medicine. There is a lack of information on the safety of medications for females who are pregnant or nursing. Providers have long adhered to the A, B, C, D, and X pregnancy grades established by the US Food and Drug Administration (FDA) [121]. Table 3 lists the medications that can be safely used or discontinued during pregnancy.

**Table 3:** Medications to continue or discontinue during pregnancy. Reproduced under the terms of the Creative Commons attribution license: A better family plan. (2007). Accessed: May 3, 2023: https://www.the-rheumatologist.org/article/a-better-family-plan/5/ [122]

Medications to continue during pregnancy	Medications to discontinue prior to pregnancy
Prenatal multivitamin	Cyclophosphamide
Low-dose aspirin	Mycophenolate mofetil
Hydroxychloroquine	Methotrexate
Prednisone (moderate dose)	Leflunomide
Azathioprine	
Aspirin (81 mg)	

Contraception and Family Planning 

Studies report that many females with SLE do not use effective contraception adequately [123,124]. Extensive contraception counseling should be provided to avoid the risks of unwanted pregnancies during periods of moderate to severe disease activity and teratogen intake. Counseling should include individualized discussions about effective available contraceptive options and should take disease-related factors into account, especially disease activity and the risk of thrombosis (presence of antiphospholipid antibodies (aPL) in particular). Table 4 illustrates the association of these factors with various contraceptive modalities.

**Table 4:** Summary of recommendations for contraceptives in SLE patients. Note: Etonogestrel implant not included due to lack of data SLE, systemic lupus erythematosus; APS, antiphospholipid syndrome; IUD, intrauterine device; LNG, levonorgestrel; DMPA, depot medroxyprogesterone acetate; COC, combined oral contraceptive; vaginal ring, combined hormonal vaginal ring; patch, combined hormonal patch; aPL, antiphospholipid antibody Reproduced under the terms of the Creative Commons attribution license: Summary of recommendations for contraceptives in SLE/APS patients. (2023). Accessed: April 28, 2023: https://www.uptodate.com/contents/image?imageKey=RHEUM%25252F98708&topicKey=RHEUM%25252F95507&search=contraception%20in%20sle&rank=1%25257E150&source=see_link [125]

Clinical presentation	Copper IUD	LNG IUD	Progesterone-only pill	DMPA	COC	Vaginal ring	Patch
SLE, low disease activity, (-) aPL	Effective, long-acting		No increase flare	Risk of osteoporosis with prolonged use	No increase flare	Similar estrogen level to COC, no data	Higher estrogen levels than COC, avoid
SLE, active disease, (-) aPL	Effective, long-acting		No increase flare	Risk of osteoporosis with prolonged use	No studies, avoid		
SLE stable on immunosuppressive medication, (-) aPL	Effective, long-acting, no infection data but likely low-risk		No increase flare	Risk of osteoporosis with prolonged use	Check for medication interactions		
SLE with renal impairment, (-) aPL	Effective, long-acting		No increase flare	Risk of osteoporosis with prolonged use	Avoid drospirenone-containing COC due to risk of hyperkalemia		
SLE with (+) aPL	Effective, long- acting	Low/no increase thrombosis	Low/no increase thrombosis	Low/uncertain risk thrombosis	Increased risk of thrombosis, avoid		
SLE with thrombosis/on anticoagulation	Increase menstrual bleeding	Low/no increase thrombosis; decreases menstrual bleeding /amenorrhea	Low/no increase thrombosis	Low/uncertain risk thrombosis	Increased risk of thrombosis, avoid		

Copper and levonorgestrel intrauterine devices (IUDs) are safe options compatible with all levels of disease activity [126] and have the advantage of providing long-term contraception. Both IUDs can also be safely used in patients with concomitant aPL. Levonorgestrel IUD has the additional benefit of controlling menorrhagia in patients who are on anticoagulation medications [127]. Previous concerns regarding the increased risk of pelvic infections with a copper IUD have not been entirely supported in many studies [128]. Moreover, immunosuppressive therapy is not a contraindication for using IUDs [129]. Other options, for the purpose of long-term contraception, include subdermal progestin-based implants. Progestin-only contraception does not increase the risk of thromboembolism as seen in a large meta-analysis study [130].

Among hormonal contraceptives, combined oral contraceptive (COC) pills, and progesterone-only pills, safety has been established for use in inactive or stable active disease with negative antiphospholipid antibodies (aPL) [126-131]. However, the use of combined hormonal contraceptive pills should be discouraged in patients with positive antiphospholipid antibodies (aPL) [126]. A case-control study found that in patients with positive aPL, COCs increased the risk of arterial thrombosis [132]. Lastly, other combined hormonal contraceptives such as vaginal rings and transdermal patches have estrogen levels either similar or more to COCs, respectively, and hence should be avoided for use in SLE [133]. The use of depot medroxyprogesterone acetate (DMPA) injections should be avoided for long-term contraception in patients being treated with corticosteroids due to the potential risk of osteoporosis [134].

Pregnancy 

The management of SLE in pregnant females is guided by the risk of disease-related complications for pregnant females and the fetus, as well as the benefits versus risks associated with different therapies available for this population.

Adverse Pregnancy Outcomes and Predictors

SLE is associated with an increased risk of obstetric complications and unfavorable pregnancy outcomes. Preeclampsia is the most frequently occurring complication among several others, including increased length of hospital stay, hypertension, intrauterine growth retardation (IUGR), preterm birth, and stillbirth [135-140]. Increased disease activity, in the preconception period, is strongly associated with adverse pregnancy outcomes including premature birth and fetal loss [141], and pregnancy should be delayed for at least six months after remission is achieved [135,141]. In addition to a high SLEDAI score, having lupus nephritis within six months before pregnancy is associated with higher rates of maternal complications including disease flares during pregnancy [142,143]. Other factors associated with adverse outcomes are major organ involvement, the presence of anti-Ro/La antibodies [144,145], and the presence of hypercoagulability [146]. Discontinuation of hydroxychloroquine is associated with the risk of disease flares during pregnancy and the postpartum period [147,148] as well as preeclampsia [149].

Management Therapies During Pregnancy and Lactation

Pregnancies were previously discouraged given the high risk of lupus flares and adverse obstetric outcomes, but advancements in medical knowledge and the development of compatible medication have made successful pregnancies possible. The teratogenicity of medication must be considered while treating pregnant patients. Table 5 summarizes the recommendations regarding the use of various medications during pregnancy and lactation period.

**Table 5:** Safety of common SLE treatments during pregnancy and lactation. SLE, systemic lupus erythematosus; NSAIDs, nonsteroidal anti-inflammatory drugs Reproduced under the terms of the Creative Commons attribution license: Dao KH, Bermas BL: Systemic lupus erythematosus management in pregnancy. Int J Womens Health. 2022, 14:199-211. 10.2147/IJWH.S282604 [150]

Medication	Preconception	During pregnancy	Lactation
Compatible			
Hydroxychloroquine	+	+	+
Sulfasalazine	+	+	+
Azathioprine	+	+	+
Cyclosporine	+	+	+
Tacrolimus	+	+	+
Prednisone	Keep dose <20 mg/day	Keep dose <20 mg/day	Keep dose <20 mg/day
NSAIDs	Discontinue with difficulty conceiving	Stop at week 20	+
Stop at conception			
Belimumab	Stop with positive pregnancy test	-	+
Rituximab	Stop with positive pregnancy test	-	+
Abatacept	Stop with positive pregnancy test	-	+

Stopping hydroxychloroquine (HCQ) precipitates flares of lupus, while continuation during the pregnancy decreases the need for average glucocorticoid dosing [147,148,151]. HCQ is known to be safe for the fetus. A case series study documented no association between HCQ and congenital abnormalities [152]. Another double-blinded placebo-controlled study documented higher delivery age and Apgar score with zero incidences of ophthalmological and auditory abnormalities in early childhood [151]. Moreover, it can be safely continued during breastfeeding [153]. According to the US Preventive Services Task Force (USPSTF) recommendation statement, low-dose aspirin should be initiated from approximately 12 weeks of gestation in females at high risk of developing preeclampsia, which includes patients with SLE [154]. The lowest possible dose of prednisone should be used to control the disease as glucocorticoids are well known to cause gestational complications such as hypertension and diabetes. While earlier studies have mentioned an increased risk of cleft palate, a large study of 51,973 infants exposed to glucocorticoids in utero did not demonstrate this association [155]. Prednisone transfer to breast milk is minimal and is considered safe for the nursing infant when taken in low doses (<20 mg/day) [156].

Nonsteroidal anti-inflammatory drugs (NSAIDs) are widely used to help control pain in patients with musculoskeletal involvement. The US Food and Drug Administration (FDA) recommends against using NSAIDs around 20 weeks of gestation due to the risk of fetal renal dysfunction leading to oligohydramnios [157,158]. NSAIDs are widely used as analgesics after parturition to treat pain in postpartum females and are generally considered safe [159]. Azathioprine use in central nervous system (CNS) disease and lupus nephritis is associated with improved survival and fewer hospitalizations [78]. A study of 178 pregnancies showed that the rate of poor pregnancy outcomes did not differ between the azathioprine-exposed and non-exposed groups, and no congenital abnormalities were noted in the infants [160]. Calcineurin inhibitors such as tacrolimus and cyclosporine are well known for achieving and maintaining remission in moderate to severe lupus, including lupus nephritis, and can be used as options where other modalities are either not tolerated or contraindicated, such as during gestation. A study of 54 pregnancies showed no difference in adverse maternal and fetal outcomes between tacrolimus-exposed and non-exposed pregnancies [161]. Subsequently, an observational study of 60 pregnancies did not demonstrate a difference in rates of fetal mortality, preterm delivery, hypertensive disorders of gestation, and small for gestational age infants in either of the groups [162]. Limited observation of cyclosporine also suggests an acceptable benefit/risk ratio for use in pregnancy [163].

Biological medications such as rituximab and belimumab are immunoglobulin G (IgG)-based medications that do not significantly cross the placenta until after 12 weeks of gestation. Hence, they can be cautiously continued through conception and should be stopped after the first missed period where possible. Alternatively, several studies [164-166] have provided enough evidence to support the continued use of biologics throughout pregnancies where alternate therapies fail to achieve disease control. Cyclosporine, tacrolimus, azathioprine, and anti-TNF-α inhibitors are also considered safe to be used during the lactation period [156].

Antineoplastic therapies and leflunomide are used for moderate to severe disease/major organ involvement but are strictly contraindicated in pregnancy [72,167-170]. The teratogenicity of CYC has been extensively studied in the past and is well known to cause fetal malformations and CNS and musculoskeletal defects [171] if used in the first trimester of pregnancy. Mycophenolate mofetil is one of the most commonly used drugs for lupus nephritis. It is not only associated with pregnancy loss [172] but is also associated with multiple fetal malformations including facial, esophageal, and ear defects [173-176]. Methotrexate is also a well-known abortifacient, hence resulting in pregnancy loss simultaneously predisposing the exposed fetus to intrauterine growth restriction and CNS, skull, and other musculoskeletal defects [177]. Leflunomide, while not yet established to be a human teratogen, is known to be embryotoxic and teratogenic in non-human models. Hence, it is classified as pregnancy category X by the FDA [178]. Leflunomide has a half-life of two weeks, and it can take a long time for levels to become undetectable in the blood after the therapy is discontinued [179]. Patients who wish to become pregnant should undergo a washout procedure with cholestyramine to aid in the elimination of the drug before conception [179,180]. Literature on CYC, mycophenolate, methotrexate, and leflunomide pertaining to breastfeeding is not sufficient to support or contradict their use during lactation. While most of the other therapies appear to be safe, these medications should not be continued during lactation [167].

Cardiovascular disease (CVD) risk in premenopausal patients with SLE

Cardiovascular disease (CVD) is one of the main causes of mortality globally [181]. Obesity, smoking, hypertension, physical inactivity, dyslipidemia, and diabetes were considered to be risk factors for CVD in females. However, in recent years, other risk factors have risen dramatically, which include preterm delivery, hypertensive disorders in pregnancy, breast cancer treatment, depression, and autoimmune diseases such as rheumatoid arthritis (RA) and SLE [182]. SLE affects every aspect of the immune system, and humoral autoimmunity with the development of specific autoantibodies and serum cytokine dysregulation are the disease hallmarks. Female bias in autoimmunity might be attributed to a variety of factors, which include the obvious sex-related variations in the immune systems of males and females that lead to females’ increased immunologic reactivity and may predispose them to acquire an autoimmune illness, possibly because estrogen inhibits Th1- dependent disorders but potentiates Th2-dependent disorders [183]. The prevalence of premature CVD in premenopausal females with SLE is estimated to be up to 50 times higher than in the general population with a male/female ratio of 9:1 [184].

The mechanism of CVD in premenopausal females with SLE is multifactorial. They are induced because of accelerated atherosclerosis, and autoimmune disorders are related to an increase in cardiovascular illnesses. These are presently regarded as nontraditional risk factors, with an increase in coronary and cerebral-vascular mortality [185]. Chronic inflammation associated with SLE leads to endothelial dysfunction, which is a key factor in the pathogenesis of CVD. This inflammation also increases the risk of atherosclerosis and promotes the development of thrombosis. It leads to the aging of vessels and raises the cumulative cardiovascular risk, just like metabolic syndrome, obesity, diabetes, chronic renal insufficiency, and sleep apnea syndrome (SAS) [185]. The recruitment of monocytes to the artery wall is an important step in the development of atherosclerosis and endothelial production of vascular cell adhesion molecule-1 (VCAM-1), which contributes to this process [186].

SLE is an inflammatory systemic illness with a typical sequela of early atherosclerosis [187]. Circulating immune complexes (IC) are also widely seen in the serum of SLE patients and are responsible for many of the disease’s acute inflammatory symptoms. Our findings that immune complex IC-C1q reduces cholesterol 27-hydroxylase mRNA and protein levels in endothelial cells and macrophages add to the theory that IC-C1q contributes to the development of atherosclerosis and may alter crucial metabolic processes in the vessel wall. Circulating IC, therefore, promotes atherosclerosis through two mechanisms, either by encouraging macrophage recruitment to the arterial wall and/or by decreasing endothelial and macrophage capacity to undertake reverse cholesterol transport by lowering intracellular cholesterol 27-hydroxylase levels. The immune response to self-antigens causes tissue damage or dysfunction in autoimmune diseases, which can occur systemically or affect specific organs or body systems. Numerous studies have linked IFN-α to SLE. During self-material uptake, IFN-α activates antigen-presenting cells, breaking immunologic self-tolerance [188]. Many SLE patients have increased serum IFN-α, which correlates with disease activity [189,190].

Recombinant human IFN-α used to treat chronic viral hepatitis and cancer may produce de novo SLE [191]. IFN-α-induced SLE usually disappears after discontinuation [192,193]. Of healthy first-degree relatives of SLE patients, 20% have excessively high serum IFN-α compared to 5% of healthy unrelated persons [194]. These findings imply that elevated serum IFN-α is heritable for SLE [194]. Patients and healthy first-degree relatives have the highest serum IFN-α activity during peak SLE incidence [195]. Polygenic inheritance may explain SLE families’ elevated IFN-α characteristics. These studies strengthen the idea that IFN-α pathway dysregulation causes human SLE. Also, there was a trend toward an inverse connection between age and serum IFN-α activity in both male and female patient groups, as well as male and female healthy relatives [195].

Managing CVD manifestations of SLE in premenopausal females

There are several therapeutic interventions that can be used to mitigate CVD manifestations in premenopausal females with SLE.

Lifestyle Modifications

Lifestyle modifications such as regular exercise, healthy diet, and smoking cessation can reduce the risk of CVD in this population [196], considering that obesity is more common among patients with SLE than in the general population [197], with prevalence ranging from 28% to 50% [198]. Established risk factors such as hypertension, dyslipidemia, and diabetes must be managed appropriately [199].

NSAIDs

Nonsteroidal anti-inflammatory drugs (NSAIDs) are commonly used to manage SLE-related symptoms and should be used cautiously in patients with a high risk of CVD. NSAIDs are used to treat fever, arthritis, serositis, and headaches in over 80% of individuals with SLE. NSAID-induced hepatotoxicity, acute renal failure, cutaneous and allergy responses, and aseptic meningitis is enhanced in SLE patients. However, NSAIDs should be safely recommended to most lupus patients if their administration is reevaluated on a frequent basis [200]. Long-term NSAID use has no elevated CVD risks, according to studies, while some studies also suggest an increased CVD risk. More research into other autoimmune/auto-inflammatory disorders is needed to see if they have similar effects [201].

Reduction of Lipids

As cellular processes require both energy and associated signaling, lipogenesis is a prime therapeutic focus. Furthermore, immunocytes rely on lipids to exert their specialized roles in response to stimuli; hence, it is important to strike a balance between the effects of lipid metabolism on immunocytes and the internal environment in order to investigate additional targets. Furthermore, several factors have been implicated in the complicated pathways of lipid metabolism that operate directly or indirectly on SLE. The importance of studying the consequences of interfering with lipid metabolism in appropriate in vivo models, in particular in combination with standard immunosuppressive medications, is emphasized by the role that lipids play in the immune system, particularly among various cell populations [202]. Thus, statins, which are lipid-lowering agents, may be used to reduce the risk of atherosclerosis in these patients, but the proven benefit still requires more data to establish the same [203].

Corticosteroids

Long-term use of corticosteroids for their anti-inflammatory and immunosuppressive effects has been a mainstay of SLE treatment for decades. Just around 10% of prednisolone and prednisone are able to penetrate the placenta because they are inactivated by 11-hydroxysteroid dehydrogenase [204]. Inflammatory rashes on the skin can be treated with topical steroids, and intralesional preparations are an option for discoid lupus that has progressed to a severe stage. Intra-articular steroid injections, with or without a local anesthetic agent, may be a more direct and preferred therapy of choice for localized soft tissue and joint involvement. Due to their pro-atherogenic qualities, corticosteroids have been linked to deleterious effects on metabolic parameters such as body fat distribution, blood pressure, and glucose metabolism [205-207]. According to research [208,209], they increase low-density lipoprotein (LDL) cholesterol and triglyceride levels while decreasing high-density lipoprotein (HDL) cholesterol. There is growing evidence that inflammation itself predisposes to atherosclerosis, and steroids can reduce this risk [206], so it is important to evaluate the benefit/risk ratio of reducing chronic inflammation versus side effects and cardiovascular risk on an individual basis. Long-term usage of corticosteroids for the treatment of SLE [210] has been linked to weight increase (from under 10 to over 30 pounds). Therefore, the increased risk of cardiovascular disease associated with the excess body weight seen in SLE patients on prolonged use of corticosteroids creates a vicious loop in which weight gain might maintain disease activity and necessitate the maintenance of these medications.

Aspirin

Aspirin therapy may also be considered in selected patients with a high risk of thrombosis. To more precisely define its function in these patients, controlled, prospective investigations are required [211].

Hydroxychloroquine

Hydroxychloroquine (HCQ), a regularly used SLE treatment, may have cardioprotective benefits and is linked to a lower incidence of CVD in these patients. HCQ may lower the incidence of flares, allowing for a decrease in steroid dosage, lessen organ damage, and prevent the thrombotic effects of antiphospholipid antibodies. The medication is generally safe and can be given to pregnant women [212].

Myasthenia gravis (MG) and SLE

SLE generally has a relapsing and remitting course, and in 90% of cases, it is seen in females of childbearing age. Its severity varies from mild to rapidly progressing, along with symptoms such as facial rash, photosensitivity, arthritis, fever, serositis, cytopenia, and oral ulcers [213]. On the other hand, myasthenia gravis (MG) is a rare organ-specific autoimmune disease of the neuromuscular junction characterized by autoantibodies against the nicotinic acetylcholine receptor (nACHR), the lipoprotein receptor-related protein 4 (LRP4), or the muscle-specific tyrosine kinase (MuSK) receptors [214]. This leads to the blockage of the neuromuscular junction resulting in muscular weakness, which improves with rest and worsens with muscle use. It generally occurs in females of age <40 years with ocular symptoms (diplopia and ptosis); slurred speech; difficulty in swallowing, chewing, walking, and lifting objects; facial weakness; and dyspnea [215]. Rarely, SLE and MG have similar features that precede one another or can coexist in a patient [214].

However, it is quite interesting to note that in patients undergoing thymectomy for MG, the association between SLE and MG has been reported. For example, a case report of a 48-year-old female stated the occurrence of SLE and secondary antiphospholipid syndrome (APS) 28 years post-thymectomy for MG, with thymectomy being the sparkling factor [216]. Thymectomy is the first-line treatment for generalized or serious myasthenia because thymic abnormalities are frequently seen in MG patients and the thymus is known to produce autoantibodies [215,217]. Thymectomy, however, has no impact in cases of SLE that have already been diagnosed [217]. A study was conducted among 13 patients with MG and preexisting SLE, out of which 11 had sufficient data available. Table 6 summarizes the diagnoses of these patients based on the standards established by the ACR [218].

**Table 6:** Common clinical characterizations in SLE and MG overlap as noted in 11 out of 13 patients. *Anti-DNA or anti-Smith, anti-cardiolipin antibodies, or lupus anticoagulant ACR, American College of Rheumatology; SLE, systemic lupus erythematosus; MG, myasthenia gravis Reproduced under the terms of the Creative Commons attribution license: Kigawa N, Pineau C, Clarke AE, et al.: Development of myasthenia gravis in systemic lupus erythematosus. Eur J Case Rep Intern Med. 2014, 1:10.12890/2014_000020 [218]

Total number	11
ACR criteria for SLE diagnosis	Number (%)
Malar rash	2 (18.2%)
Oral ulcers	2 (18.2%)
Photosensitivity	2 (18.2%)
Renal disorder	2 (18.2%)
Discoid rash	3 (27.3%)
Neurological disorder	4 (36.4%)
Serositis	6 (54.5%)
Hematological disorder	8 (72.7%)
Arthritis	10 (90.9%)
Immunologic disorder*	10 (90.9%)
Antinuclear antibody	11 (100%)

Immunologic Link in the Pathogenesis

The pathogenesis of both conditions has been linked to an alpha-chemokine subfamily (CXC) in particular [219,220]. According to studies, these chemokines are in charge of several immunoreactive cell mobility via chemoattraction. Additionally, they are involved in angiogenesis and may facilitate the activation of dendritic cells, monocytes, T cells, B cells, NK cells, basophils, and eosinophils [221]. Studies on animal models have demonstrated that CXCL13 interacts with B and T lymphocytes, causing the precipitation of SLE in patients with established MG [219]. Granulocyte-macrophage colony-stimulating factor (GM-CSF), which can be found exogenously and endogenously, is also a contributing component in the development of both diseases. It is worth noting that, in addition to vascular endothelial cells, fibroblasts, mast cells, monocytes, and macrophages, T and B cells are all involved in the endogenous synthesis of GM-CSF, indicating its significant immunologic association [222].

Thymectomy and the Increasing Risk for Autoimmune Diseases

The thymus is where T cells mature. Pathological processes in the thymus induce cell dysfunction and activate autoreactive CD4+ T lymphocytes, which interact with B lymphocytes to produce autoantibodies [223]. By suppressing the activity of CD4+ T lymphocytes, regulatory T lymphocytes are in charge of stopping the autoimmune process. It has been suggested that the absence or dysfunction of regulatory CD4+ CD25+ T lymphocytes is a cause of connective tissue disorders and consequently of MG and SLE [223]. The occurrence of systemic autoimmune disorders in patients who underwent thymectomy for MG has been documented in some situations [217]. Loss of central tolerance to its antigen and increased autoantibody production are side effects of thymectomy [224]. Polyarthritis and polyarthralgia were the most prevalent symptoms in post-thymectomy SLE cases, and laboratory results revealed mild T-cell lymphopenia, hypergammaglobulinemia, and B-cell hyperreactivity [225]. Many autoimmune illnesses, including SLE, Hashimoto’s disease, APS, idiopathic portal hypertension, and cutaneous vessel vasculitis, can be made worse by thymectomy [225]. Table 7 displays the prevalence and concomitance of SLE and MG as reported in various articles [224,226-228].

**Table 7:** Prevalence and concomitance of SLE and MG. n, total number; Av, average; M, male; F, female; MG, myasthenia gravis; SLE, systemic lupus erythematosus Reproduced under the terms of the Creative Commons attribution license: Tanovska N, Novotni G, Sazdova-Burneska S, et al.: Myasthenia gravis and associated diseases. Open Access Maced J Med Sci. 2018, 6:472-8. 10.3889/oamjms.2018.110 [226], Bekircan-Kurt CE, Tuncer Kurne A, Erdem-Ozdamar S, Kalyoncu U, Karabudak R, Tan E: The course of myasthenia gravis with systemic lupus erythematosus. Eur Neurol. 2014, 72:326-9. 10.1159/000365568 [227], and Sthoeger Z, Neiman A, Elbirt D, et al.: High prevalence of systemic lupus erythematosus in 78 myasthenia gravis patients: a clinical and serologic study. Am J Med Sci. 2006, 331:4-9. 10.1097/00000441-200601000-00004 [228]

SN	MG (n)	SLE (concomitant)	Gender (M:F)	Age at diagnosis (Av)	Thymectomy associated with SLE	Prevalence	Reference
1	127	1	0:01	48.8	-	0.80%	[14]
2	132	5	1:04	24-58	2/5	3.78%	[15]
3	78	6	0:06	44.5	2/6	7.70%	[16]
SN	SLE (n)	MG (concomitant)	Gender (M:F)	Age at diagnosis (Av)	-	Prevalence	-
1	1300	17	0:17	34.5	-	1.30%	[12]

Although it was believed that HCQ can lead to the development of MG, patients who received this medication for SLE displayed weaker MG symptoms than other patients [224,226]. Antimalarial medications directly impact the neuromuscular junction, frequently leading to neuromyopathy and atrophic muscle fibers in muscle biopsies [224]. When symptoms persist even after discontinuing HCQ, MG should be ruled out because the drug can cause ocular symptoms and symmetrical muscle weakness [214].

Simultaneous Management of Both Diseases

Four patients with SLE-MG overlap syndrome were reported in a case series; two underwent thymectomy after the diagnosis of MG was established and received pyridostigmine, while the third case initially responded poorly to pyridostigmine and was later identified as having SLE-myositis overlap syndrome [217]. This series shows various treatment plans for different cases. For the case series reported by Minchenberg et al. [217], the events and the treatment are shown in Table 8.

**Table 8:** A case series of four patients who underwent thymectomy and other treatment strategies for SLE and MG overlap management. M, male; F, female; MG, myasthenia gravis; SLE, systemic lupus erythematosus; APS, antiphospholipid syndrome; HCQ, hydroxychloroquine Reproduced under the terms of the Creative Commons attribution license: Minchenberg SB, Chaparala G, Oaks Z, Banki K, Perl A: Systemic lupus erythematosus-myasthenia gravis overlap syndrome: presentation and treatment depend on prior thymectomy. Clin Immunol. 2018, 194:100-4. 10.1016/j.clim.2018.07.007 [217]

Variables/findings	Case 1	Case 2	Case 3	Case 4
Age/gender	56/F	57/M	58/F	62/F
Age at MG onset	10	54	58 (initially diagnosed as SLE)	33
SLE	Present	Present	Present	Present
Thymectomy	Yes	No	No	Yes
Treatment (initial)	Thymectomy, pyridostigmine	Cholinesterase inhibitor	HCQ (discontinued by personal decision)	Thymectomy, pyridostigmine
Treatment (later)	HCQ	Mycophenolate mofetil, HCQ	None	HCQ

**Figure 9:**
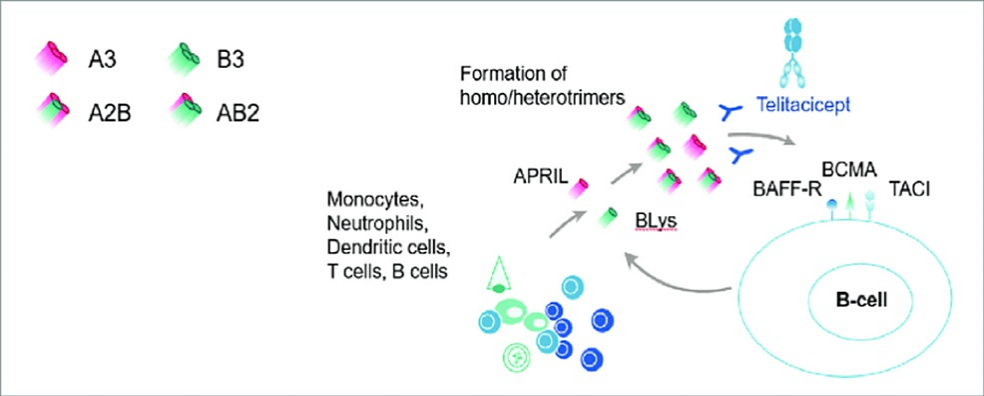
Mechanism of action of telitacicept. Telitacicept binds to a BLyS and APRIL and thereby prevents them from binding to BAFF-R, BCMA, and TACI receptors expressed on the B-cell surface, suppressing BLyS and APRIL signaling and inhibiting the development and survival of mature B cells and plasma cells. A3, APRIL homotrimers; B3, BLyS homotrimers; A2B, heterotrimers of two APRIL and one BLyS molecules; AB2, heterotrimers of one APRIL and two BLyS molecules BLyS, B lymphocyte stimulator; APRIL, a proliferation-inducing ligand; BAFF-R, B-cell activating factor receptor; BCMA, B-cell maturation antigen; TCAI, transmembrane activator and calcium modulator and cyclophilin ligand interactor Reproduced under the terms of the Creative Commons attribution license: Fan Y, Gao D, Zhang Z: Telitacicept, a novel humanized, recombinant TACI-Fc fusion protein, for the treatment of systemic lupus erythematosus. Drugs Today (Barc). 2022, 58:23-32. 10.1358/dot.2022.58.1.3352743 [230]

Telitacicept (Tai'ai®) is a fusion protein made up of a recombinant transmembrane activator, calcium modulator, and cyclophilin ligand interactor (TACI) receptor fused to the fragment crystallizable (Fc) region of human immunoglobulin G (IgG). For the therapy of B-cell-mediated autoimmune conditions such as SLE, rheumatoid arthritis (RA), and multiple sclerosis (MS), Yantai Rongchang Pharmaceutical is developing telitacicept through its subsidiary RemeGen. Telitacicept inhibits the growth and survival of mature B cells and plasma cells by binding to and inhibiting the action of two cell-signaling molecules, B lymphocyte stimulator (BLyS) and a proliferation-inducing ligand (APRIL). The use of telitacicept for the treatment of patients with active SLE was given its initial permission in China in March 2021. China is currently conducting clinical trials of telitacicept for a number of additional indications, such as IgA nephropathy, MS, MG, neuromyelitis optica, spectrum diseases, RA, and Sjögren’s syndrome. The development of telitacicept, which resulted in this first approval for SLE, has been summarized in this paper [229]. Figure 9 shows the mechanism of action of telitacicept.

Managing antiphospholipid antibody syndrome (APS) with SLE

Antiphospholipid syndrome (APS) can be defined as the association of thrombosis and/or pregnancy morbidity with antiphospholipid antibodies (aPL), lupus anticoagulant (LA), anticardiolipin antibodies (aCL), and/or anti-β2-glycoprotein-I antibodies (aβ2GPI) [231]. APS can either be primary, which occurs in otherwise healthy individuals without an underlying disease, or it can occur simultaneously with autoimmune diseases, particularly SLE. Patients with SLE and APS can develop valvular heart disease, pulmonary hypertension (PH), livedo reticularis (LR)/racemosa, thrombocytopenia, hemolytic anemia, renal thrombotic microangiopathy (TMA), and cognitive dysfunction [232]. SLE and aPL-positive patients can both progress to end-organ damage with one-third of SLE patients progressing to end-organ damage within five years of diagnosis and one-third of aPL patients progressing to end-organ damage within 10 years of disease [233]. Around 30%-40% of patients with SLE test positive for aPL [234]. The risk of vascular events and death in SLE increases due to aPL [235,236]. Nearly 40% of aPL-positive patients among all SLE patients develop arterial and/or deep venous thrombosis (DVT) as compared to 10%-20% of aPL-negative patients [237]. Wahl et al. [238] conducted a meta-analysis that showed that patients with SLE and LA are at six times greater risk of developing venous thromboembolism and at 11 times greater risk of developing recurrent venous thromboembolism as compared to those without LA. Patients having SLE with aPL are twice at risk of developing DVT/pulmonary embolism (PE) and four times at risk of developing recurrent DVT after the first event as compared to those without aCL.

Pregnancy morbidity in patients with SLE and aPL is 25%-47%, while that in SLE without aPL is 0%-38% [239,240]. In patients with lupus nephritis, aPL increases the risk of maternal hypertension and premature births. aPL is also associated with an increased rate of induced abortion [241]. The presence of valvular lesions is 40%-50% in aPL-positive lupus, while it is around 20% in aPL-negative SLE [242]. The frequency of pulmonary hypertension in patients with SLE and aPL is 15%-100%, and in SLE without aPL, it is 11%-55% [243]. A meta-analysis conducted by DeFilippis et al. [244] showed that the percentage of livedo reticularis in aPL-positive SLE was 27%, and in aPL-negative SLE, it was 11%. SLE patients with LA or aCL are three times more likely to have thrombocytopenia as compared to those without LA or aCL, as shown by a meta-analysis conducted by Love et al. [237]. Autoimmune hemolytic anemia is found in 20%-28% of aPL-positive SLE, while its percentage is 1%-9% in aPL-negative SLE patients [245]. aPL nephropathy is present in 25%-39% of aPL-positive SLE patients, and its prevalence in aPL-negative SLE is 4%-16% [245]. A study conducted by Coín et al. [246] showed the prevalence of cognitive impairment in aPL-positive SLE patients to be 21% as compared to 11.9% in aPL-negative SLE patients.

In the majority of the cases, there is no difference in the management of aPL-positive patients with or without lupus. There are however certain exceptions. Multiple studies on aPL-positive patients with or without systemic autoimmune diseases suggested that low-dose aspirin may play a role in protecting against the first episode of thrombosis in aPL-positive SLE patients [247-249]. The use of low-dose aspirin in the first pregnancy in a patient with aPL-positive SLE is justified as both pregnancy and SLE are risk factors for thrombosis and low-dose aspirin may decrease the risk of preeclampsia in high-risk patients [245]. A few case reports have pointed out that adding warfarin, heparin, or aspirin to the standard treatment in patients with aPL nephropathy is beneficial [250-253]. The European League Against Rheumatism and European Renal Association-European Dialysis and Transplant Association (EULAR/ERA-EDTA) suggested the use of hydroxychloroquine and/or antiplatelet/anticoagulant for lupus patients with aPL nephropathy [254]. A recent study suggested that lupus patients with APS had higher activation of the mTOR pathway as compared to those lupus patients without APS. For patients with aPL nephropathy who required kidney transplantation, those who were treated with rapamycin (10 patients) had decreased vascular proliferation, and no recurrence of vascular lesions proliferation was observed. At 144 months post-transplantation, seven of 10 (70%) aPL nephropathy patients treated with rapamycin had a functioning allograft in comparison with only three of 27 (11%) patients who were not treated with rapamycin [255]. The use of low-dose aspirin remains controversial for primary thrombosis and pregnancy morbidity prevention and so is the use of anticoagulation in lupus nephritis patients with aPL nephropathy [245].

Non-pharmacological interventions for the improvement of fatigue, depression, pain, and quality of life (QOL) in SLE patients

**Figure 10:**
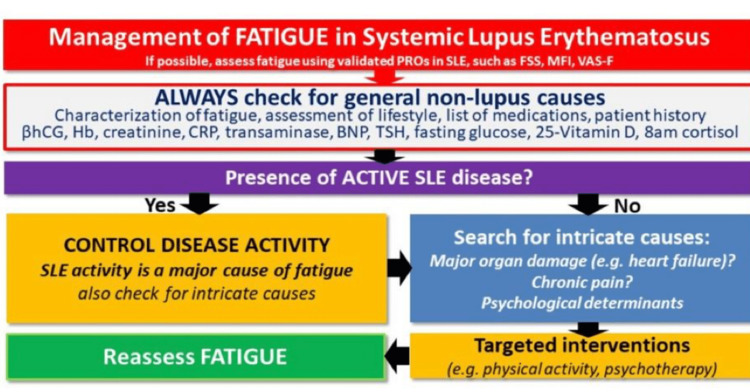
Practical algorithm for the management of fatigue in patients with SLE. SLE, systemic lupus erythematosus; PROs, patient-reported outcomes; βhCG, β-human chorionic gonadotropin; Hb, hemoglobin; CRP, C-reactive protein; BNP, brain natriuretic peptide; TSH, thyroid-stimulating hormone; FSS, fatigue severity scale; VAS, visual analog scale Reproduced under the terms of the Creative Commons attribution license: Mertz P, Schlencker A, Schneider M, Gavand PE, Martin T, Arnaud L: Towards a practical management of fatigue in systemic lupus erythematosus. Lupus Sci Med. 2020, 7:10.1136/lupus-2020-000441 [257]

Fatigue, pain, and depression significantly influence the overall quality of life of SLE patients, in conjunction with disease activity. Therefore, it is imperative to implement effective interventions aimed at improving the quality of life (QOL) for these individuals. In a literature review published in 2016, a total of 12 randomized studies with 846 participants, comprising seven randomized trials, one non-randomized trial, and four prospective observational studies, were analyzed. The review concluded that physical exercise, behavioral and psychological interventions, acupuncture, dietary modifications, and phototherapy demonstrated efficacy in mitigating fatigue among SLE patients. Notably, aerobic exercise emerged as the most effective intervention, although the study findings were not always uniform [256]. A strategy was suggested to assess and handle fatigue among SLE patients. The proposed approach entails examining potential non-lupus factors that could contribute to fatigue, monitoring disease activity, evaluating for anxiety and depression, and administering suitable interventions such as managing non-lupus etiologies, treating active disease, and utilizing medication and psychological interventions to manage anxiety and depression [257]. Figure 10 shows the management of fatigue in SLE.

Another approach to manage fatigue, exhaustion, and pain is balneotherapy. It is a therapeutic approach that utilizes the healing properties of mineral waters, mud, and natural gases from springs that are widely acknowledged and accepted for their medicinal and legal benefits. The main objective of balneotherapy is to provide a rehabilitative and restorative effect on the body. According to the research findings, incorporating balneotherapy as a supplementary component to non-pharmacological treatments may potentially result in advantageous outcomes for individuals with SLE who are in remission or have minimal disease activity. Specifically, it may help to alleviate non-inflammatory pain and fatigue, thereby enhancing the QOL for these patients [258]. Physical exercise is an essential component of managing SLE, as it can effectively reduce fatigue and depression and enhance the overall QOL. In a study conducted on 20 patients diagnosed with SLE and 25 control patients, both groups underwent a three-month strengthening exercise program. The participants’ depression level was assessed using the self-rating depression scale, QOL was measured using a questionnaire, and the severity of fatigue was assessed using the fatigue severity scale. The study also utilized the six-minute walk test, two-minute step test, and body mass index (BMI) to evaluate the participants’ physical health before and after the three-month exercise program [259].

The inclusion of a psychoeducational program in the management of SLE patients resulted in an improvement in depression, anxiety, perceived stress, QOL, satisfaction with treatment, and medication adherence but did not have an impact on disease activity, according to a study that enrolled 80 SLE patients divided equally into intervention and control groups. The intervention group received 12 group sessions of psychotherapy and patient education, while disease activity was evaluated using the SLEDAI. Various questionnaires were administered, including the Symptom Checklist-90-Revised (SCL-90-R), perceived stress scale (PSS), Short Form 36 (SF36), Treatment Satisfaction Questionnaire for Medications (TSQM), and Medication Adherence Rating Scale-5 (MARS-5) [260].

Further research studies should be conducted on non-pharmacological interventions to enhance the QOL of SLE patients. These studies should involve larger sample sizes to increase statistical power and longer follow-up periods to obtain more comprehensive results.

## Conclusions

Systemic lupus erythematosus (SLE) is a complex autoimmune disease that requires individualized management. ANA positivity is the current qualifying factor for SLE criteria, but the diagnosis should be made through an individualized approach to avoid excluding potential patients from appropriate therapies. Females of reproductive age with SLE require careful management of fertility, pregnancy, and drug safety. Chronic inflammation associated with SLE leads to endothelial dysfunction, hastening vessel aging and raising cumulative cardiovascular risk. Thymectomy in myasthenia gravis (MG) patients has been associated with an increased risk of developing autoimmune diseases such as SLE, but it has no impact on cases of SLE that have already been diagnosed. The association between SLE and MG suggests a potential role for hydroxychloroquine (HCQ) in alleviating MG symptoms in SLE patients. Antiphospholipid syndrome (APS) is associated with an increased risk of thrombosis, pregnancy morbidity, and end-organ damage in SLE patients. Low-dose aspirin can protect against thrombosis in aPL-positive SLE patients. Non-pharmacological interventions such as exercise, psychotherapy, and balneotherapy can improve the quality of life of SLE patients. Optimal outcomes for SLE patients require a multidisciplinary approach involving rheumatologists, obstetricians, and other specialists.
